# Tumour Growing from the Uterus, &c.

**Published:** 1829-01-01

**Authors:** 


### WORCESTER INFIRMARY.

### TUMOUR GROWING FROM THE UTERUS, AND PASSING BETWEEN THE SACRUM AND RECTUM.

A woman, 53 years of age, was admitted into the above Institution, in the month of August, 1813, with an ulcer on the left leg, and enlargement of the abdomen, from which she had suffered for several years.

"It was as prominent as in the sixth month of pregnancy. The enlargement was general over the lower part of the abdomen, not greater on one side than the other, extending from the pubes nearly up to the umbilicus. She was generally in pain about these parts, and in the groins; there was likewise considerable tenderness, on pressure. The appetite was good, but she was troubled with wind on the stomach. The bowels were always costive. She had frequent micturition, and always pain in evacuating the bladder. She was quite incapable of any active employment, but able to gain her livelihood by knitting, though she could not sit long in one place, being easier for gentle exercise. The breath was always short, but there was no cough. She had never borne children. The catamenia flowed till the usual period. The leg was cured by the latter end of December, and she went away from the Infirmary, much in the same state as when admitted, with respect to her visceral disease."

In September, 1815, the patient was again admitted, with an ulcer on the other leg. The belly was increased in size—the pain and tenderness were greater—the costiveness more obstinate—micturition more painful—and a thick, white, and profuse discharge incessantly flowed from the vagina. She was ordered three grains of the sulphate of iron, and ten of gum olibanum twice daily, with the effect of diminishing in the first instance, and afterwards nearly arresting the discharge from the vagina. The other symptoms, however, continued unabated, and Oct. 28, she complained of much pain in the head, and was rather delirious. The abdomen was also more tender and painful. On the 30th, the abdominal tenderness and tension were increased, the pulse was hard and wiry, and an umbilical hernia was discovered on examination. Ten leeches were applied to the abdomen, and afterwards a blister, but the patient now rapidly sank, and died at three o'clock in the morning of the 31st.

*Dissection.* On opening the abdomen, extensive inflammation of the peritoneum was discovered; the intestines were covered with purulent matter; the stomach was adherent to the liver, and the latter to the diaphragm. The liver itself was double its natural size; the spleen was much enlarged and softened in its structure, and its peritoneal coat had become cartilaginous in parts. The hernial sac at the navel, contained only omentum, and this was not strangulated.

The uterus appeared to occupy the space in the abdomen, which it does in the seventh month of utero-gestation. It was closely connected in front with the peritoneal lining of the abdominal muscles; behind, with the peritoneal covering of the bowels, whilst the round and broad ligaments connected it, as usual, with the sides of the pelvis and pudenda. On removing these connexions, and raising the organ, a tumour was observed descending from its lower and posterior part, between the rectum and os sacrum, and separating the former from the latter. The surface of this tumour was very much inflamed, and covered with purulent secretion. The vagina and urethra were naturally situated, the colon and cæcum were larger than natural, the rectum and small intestines shewed nothing unusual. On macerating the diseased parts, it was found that a tumour had grown within the uterus, resembling the muscular structure of that organ. The uterus could, with care, be separated from the tumour, to which it was attached by cellular substance; both uterus and tumour together weighed 14lbs. 8oz. and a half.

Dr. Hastings, who relates the case, considers it remarkable that the patient should suffer so little, whilst two important organs were diseased. We cannot view the matter in quite so remarkable a light. The enlargement of the liver, slow in its march, as it surely must have been, and apparently unattended with disorganization of its structure, was not an affection likely to produce any serious or characterestic symptoms in its progress. The uterine disease, on the other hand, we conceive to have presented all the indications, that a knowledge of its functions, and the fact of its not being an absolutely *vital* organ, would lead us to expect.

"Dr. Baillie seems to regard growths of this description, in the uterus, as tubercles: he says, 'A mass of the same kind is sometimes found in the cavity of the uterus, and often grows to a very large size. I have seen it a good deal larger than a child's head at birth. This mass, when cut into, exhibits precisely the same appearances as those which we have so lately described. It is remarkable that such masses within the cavity of the uterus commonly do not adhere in any part closely to it, but are connected with it loosely, by the intervention of cellular membrane and small blood-vessels, so that they can be very easily peeled off without injuring the structure of the uterus.'

"'These tubercles,' he says, in another place, 'have a structure much resembling that of the uterus itself.'*

"The size of the tumour, in the case above related, is greater than any one alluded to in Baillie's Works, as he mentions one the size of a child's head at birth, as the largest he had seen, which falls far short of the one here detailed."

The case and dissection possess much interest; especially in these uterus amputating days. To borrow an American phrase, we "guess" that the tumour passing down between the sacrum and vagina, would rather have embarrassed an operator.—MIDLAND REPORTER.

---

### XLVI.

* "Works of Dr. Baillie, by Wardrop, vol. II. p. 325."

